# Treatment of moderate to severe respiratory COVID-19: a cost-utility analysis

**DOI:** 10.1038/s41598-021-97259-7

**Published:** 2021-09-07

**Authors:** Stephen E. Congly, Rhea A. Varughese, Crystal E. Brown, Fiona M. Clement, Lynora Saxinger

**Affiliations:** 1 Division of Gastroenterology and Hepatology, Department of Medicine, Cumming School of Medicine, University of Calgary, 6th Floor, Teaching Research and Wellness Building, 3280 Hospital Drive NW, Calgary, AB T2N 4N1 Canada; 2 O’Brien Institute of Public Health, University of Calgary, Calgary, AB Canada; 3 Division of Pulmonary Medicine, Department of Medicine, University of Alberta, Edmonton, AB Canada; 4 Division of Pulmonary, Critical Care and Sleep Medicine, University of Washington, Seattle, WA USA; 5 Department of Community Health Sciences, University of Calgary, Calgary, AB Canada; 6 Division of Infectious Diseases, Department of Medicine, University of Alberta, Edmonton, AB Canada

**Keywords:** Respiratory tract diseases, Infectious diseases, Antimicrobial therapy, Health care economics

## Abstract

Despite COVID-19’s significant morbidity and mortality, considering cost-effectiveness of pharmacologic treatment strategies for hospitalized patients remains critical to support healthcare resource decisions within budgetary constraints. As such, we calculated the cost-effectiveness of using remdesivir and dexamethasone for moderate to severe COVID-19 respiratory infections using the United States health care system as a representative model. A decision analytic model modelled a base case scenario of a 60-year-old patient admitted to hospital with COVID-19. Patients requiring oxygen were considered moderate severity, and patients with severe COVID-19 required intubation with intensive care. Strategies modelled included giving remdesivir to all patients, remdesivir in only moderate and only severe infections, dexamethasone to all patients, dexamethasone in severe infections, remdesivir in moderate/dexamethasone in severe infections, and best supportive care. Data for the model came from the published literature. The time horizon was 1 year; no discounting was performed due to the short duration. The perspective was of the payer in the United States health care system. Supportive care for moderate/severe COVID-19 cost $11,112.98 with 0.7155 quality adjusted life-year (QALY) obtained. Using dexamethasone for all patients was the most-cost effective with an incremental cost-effectiveness ratio of $980.84/QALY; all remdesivir strategies were more costly and less effective. Probabilistic sensitivity analyses showed dexamethasone for all patients was most cost-effective in 98.3% of scenarios. Dexamethasone for moderate-severe COVID-19 infections was the most cost-effective strategy and would have minimal budget impact. Based on current data, remdesivir is unlikely to be a cost-effective treatment for COVID-19.

## Introduction

Severe acute respiratory syndrome coronavirus 2 (SARS-CoV-2) was first reported to cause respiratory illness in China in December 2019, with the disease designated as COronaVIrus Disease 2019 (COVID-19)^1^. SARS-CoV-2 has caused significant morbidity and mortality globally; as of May 21, 2021, there have been 165,705,287 documented cases worldwide and 3,434,082 confirmed deaths with the highest national burden thus far in the United States^2^. Given this impact, there has been great interest in finding potential treatments for COVID-19^3^ although early enthusiasm and adoption of possible candidate drugs, such as hydroxychloroquine, has been tempered upon rigorous study^4^.

Currently, two drugs have shown benefit in randomized controlled trials. First, remdesivir, a RNA polymerase inhibitor with broad antiviral activity and in vitro effect against SARS-CoV-2^3^, was shown to have benefit in a randomized controlled trial in the United States sponsored by the National Institute of Health^5^ with a list price of $390 US dollars per day^6^. More recently, dexamethasone was shown to improve COVID-19 outcomes in a randomized controlled trial in the United Kingdom^7^ with a list price of approximately $20 per treatment course^8^. Complete 28-day mortality data suggests that the benefit of antiviral therapy with remdesivir is highest in patients needing supplemental oxygen with a 32% relative risk of mortality compared to placebo and a reduced length of stay^5^, while the anti-inflammatory effect of dexamethasone appears somewhat beneficial in patients on oxygen, reducing mortality by 3.5%, and most beneficial in those requiring mechanical ventilation with a reduction of mortality by 11.7%^7^. Despite the public health emergency with COVID-19, health care systems continue to need to operate within a budget and make resource allocation decisions. As such, given this and the burden of COVID-19 in the United States, we developed a cost-effectiveness analysis of remdesivir and dexamethasone in the United States context with additional global considerations assessed by willingness-to-pay thresholds.

## Methods

### Model design

**Figure 1:**
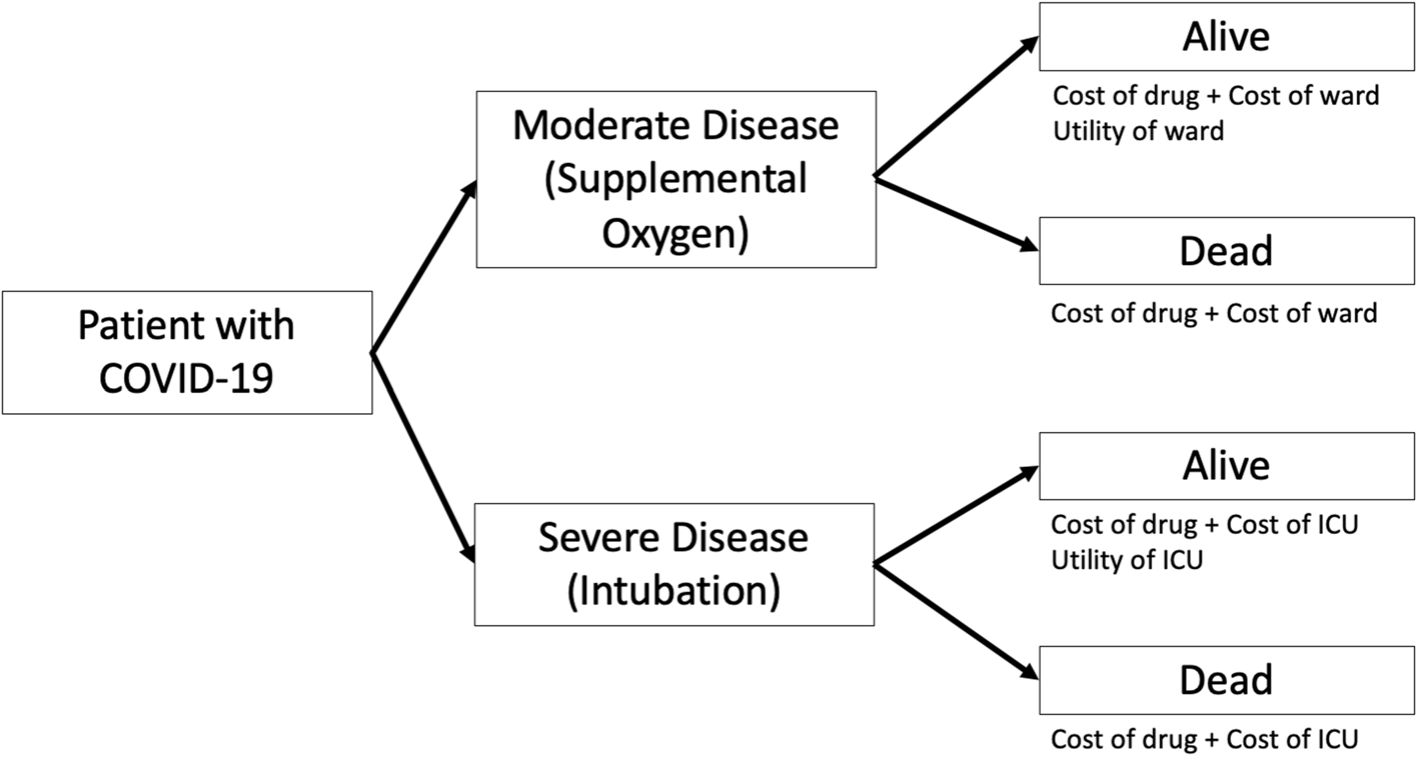
Schematic of cost-effectiveness decision tree for all treatment strategies evaluated.

A decision tree was developed with TreeAge Pro 2020 (TreeAge Software, Williamstown MA) to simulate patients with moderate-severe COVID-19 respiratory infections. The base-case scenario was a 60-year-old patient admitted to hospital with a respiratory COVID-19 infection. Cases could either be admitted to the ward on oxygen (moderate COVID-19 infection) or the intensive care unit (ICU) (severe COVID-19 infection). The strategies compared were chosen based on current published data: giving dexamethasone to all patients, dexamethasone to only severe COVID-19 infections, remdesivir to all patients, remdesivir to moderate COVID-19 infections only, remdesivir to only severe COVID-19 infections, remdesivir to moderate and dexamethasone to severe COVID-19 infections, and best supportive care to all patients. For patients receiving dexamethasone, they were assumed to receive 6 mg per day for 10 days^7^. For patients receiving remdesivir for severe COVID-19 infections, they were assumed to receive 200 mg on day 1 and then 100 mg daily for 9 additional days. For moderate COVID-19 infections, patients were assumed to receive a 5 day course of remdesivir; 200 mg on day 1 and then 100 mg daily for 4 additional days given data suggesting similar efficacy of 5 and 10 day courses in this population^9^. In this model, a patient would either recover from their infection and survive or die with no transition between ward and ICU given that data regarding the probability of transitioning between the ICU and ward, and vice versa are not well established. A schematic of the decision tree is found in Fig. 1. To account for clinical situations in which patients may be admitted to the ICU on high flow oxygen, a scenario analysis was performed where all patients were assumed to be admitted to the ICU with the expected increase in costs. It was assumed that beyond 28 days, there would be no ongoing conditions expected to significantly impact assessed quality adjusted life years for the rest of the one-year time-horizon.

### Data sources

Data from a meta-analysis of the efficacy of remdesivir in COVID-19^10^ as well as randomized control trial data for dexamethasone^7^ populated the model; there is no published meta-analysis of dexamethasone currently and meta-analysis of the efficacy of systemic steroids^11^ are predominantly informed by the dexamethasone randomized control data used in this model. Hospital costs were based on the 2020 Medicare national payment rate^12^ for the appropriate diagnosis related group (DRG) code with moderate COVID-19 respiratory infections being classified as DRG 178 and severe COVID-19 infections classified as DRG 207. Physician costs were not included in this model given uncertainty regarding average length of stay with COVID-19. Prices are based on the 2020 Medicare Part B database^8^ for dexamethasone and the published price for remdesivir^6^. Utilities were based on previous experiences with H1N1 and influenza^13,14^; patients were assumed to have these utilities for 28 days based on their initial degree of disease severity and would not change during this period and then return to the US average utility of 0.815^15^ for the remainder of the year if they survived. Costs are presented in 2020 US dollars. Model inputs are listed in Table 1.

**Table 1:** Model inputs.

Variable	Variable	Range tested	Distribution for multivariate analysis	References
Probability of having severe COVID-19	0.15	0.05–0.25	Beta	^5,7^
Probability of death with severe COVID-19	0.25	0.15–0.45	Beta	^5,7^
Probability of death with moderate COVID-19	0.07	0.04–0.20	Beta	^5,7^
Risk reduction of death with dexamethasone for severe COVID-19	0.65	0.5–0.82	Log-normal	^7^
Risk reduction of death with dexamethasone for moderate COVID-19	0.8	0.70–0.92	Log-normal	^7^
Risk reduction of death with remdesivir for severe COVID-19	1.19	0.98–1.46	Log-normal	^10^
Risk reduction of death with remdesivir for moderate COVID-19	0.81	0.68–0.96	Log-normal	^10^
Cost dexamethasone for 10-day course at 6 mg/day	19.20	15–30	Gamma	^8^
Cost remdesivir vial	390	100–450	Gamma	^6^
Cost severe COVID-19 admission [USD] [DRG 207]	33,247.15	14,400–50,000	Gamma	^12^
Cost moderate COVID-19 admission [USD] [DRG 178]	7206.95	5020.46–10,962.59	Gamma	^12^
Utility severe COVID-19	0.23	0.18–0.28	Beta	^14^
Utility moderate COVID-19	0.5616	0.3846–0.6925	Beta	^13^

### Methodology

The perspective of the health care payer was utilized with a willingness to pay threshold of $100,000/quality adjusted life year (QALY)^16^. A 1-year time horizon was used in this model; discounting was not done due to the short time frame. Univariate analysis and a multivariate probabilistic sensitivity analysis using 100,000 second order samples were performed to assess the model; distributions used are found in Table 1. The model was internally validated and was determined to have good face validity. We followed the CHEERS checklist when writing our manuscript^17^ and our model was in keeping with best practice guidelines^18^. Ethics approval was not required as the data used in this study came from publicly available data.

### Role of the funding source

This study was unfunded. The corresponding author had full access to all the modelling data and output in the study and had final responsibility for the decision to submit for publication.

## Results

In the base-case model (Table 2), supportive care for moderate-severe COVID-19 had a cost of $11,112 for 0.7155 quality adjusted life years (QALY) per person over the one-year time horizon. Using dexamethasone for all patients was associated with an incremental cost-effectiveness ratio (ICER) of $981/QALY versus supportive care and an ICER of $1724/QALY as compared to using dexamethasone in only severe COVID-19 cases. All of the remdesivir monotherapy strategies (using it for all patients, only severe cases and only moderate cases) were both less effective and more costly than dexamethasone-based strategies (dominated). Using a severity stratified treatment approach of remdesivir for moderate COVID-19 infections and dexamethasone for severe infections was also dominated by the dexamethasone strategies.

**Table 2:** Base case analysis referencing supportive care as baseline.

Strategy	Cost [US$]	Incremental cost [US$]	Efficacy [QALY]	Incremental efficacy [QALY]	Incremental cost effectiveness ratio [US$/QALY]
Supportive care	11,112.98	–	0.7155	–	–
Dexamethasone severe	11,115.86	2.88	0.7256	0.0101	284.93
Dexamethasone all	11,132.18	19.20	0.7351	0.0196	980.84
Remdesivir severe	11,756.48	643.50	0.7100	− 0.0055	DOMINATED
Remdesivir moderate	13,101.98	1989.00	0.7245	0.0090	DOMINATED
Remdesivir moderate, dexamethasone severe	13,104.86	1991.88	0.7346	0.0191	DOMINATED
Remdesivir all	13,745.48	2632.50	0.7190	0.0035	DOMINATED

In the scenario analysis where all patients were assumed to be admitted to the ICU, supportive care now cost $33,247 and resulted in 0.7155 QALY per person per year. The incremental cost-effectiveness ratios and rankings remained unchanged from the base case except that each strategy cost an additional $22,134 accounting for the additional costs of ICU admission as compared to admission to the ward. A second scenario analysis in which hospital costs were reduced by 60% to try and extrapolate these findings outside of the United States context led to supportive care costing $6668 per 0.7155 QALY per person per year and no change in the incremental cost-effectiveness ratios and rankings from the base case.

In univariate sensitivity analysis, the preferred strategy of dexamethasone for all patients remained unchanged with all variables. The remdesivir for moderate and dexamethasone for severe COVID-19 infections strategy with variation of the risk reduction of dexamethasone for moderate COVID-19 or the risk reduction of remdesivir for moderate COVID-19 no longer was less effective than the dexamethasone for all strategy, and as such was no longer dominated. At the extremes of sensitivity tested, ICER values were over $275,000 as compared to the reference of supportive care and would not be the preferred strategy (Table 3). In an exploratory threshold analysis, remdesivir for moderate and dexamethasone for severe COVID-19 infections became less costly than dexamethasone for all (and no longer was dominated) if remdesivir cost < $3.17 per dose which is an unlikely price.

**Table 3:** Univariate sensitivity analysis thresholds.

Variable	Base case (range tested)	Threshold	Impact	Incremental cost-effectiveness ratio (vs. standard of care)
Risk reduction of dexamethasone for moderate COVID-19 infection	0.8 (0.70–0.92)	> 0.802	Remdesivir for moderate and dexamethasone for severe COVID-19 infections no longer dominated	ICER at 0.92 = $347,284/QALY
Risk reduction of remdesivir for moderate COVID-19 infection	0.81 (0.68–0.96)	< 0.812	Remdesivir for moderate and dexamethasone for severe COVID-19 infections no longer dominated	ICER at 0.68 = $277,827/QALY

Probabilistic sensitivity analysis of all strategies based on a willingness to pay threshold of $100,000 showed that in 98.26% of simulations, using dexamethasone for all patients was the favoured strategy while using remdesivir in moderate and dexamethasone in severe COVID-19 infections would be favoured in 1.66% of simulations. Dexamethasone for only severe COVID-19 infections was favoured in 0.007% and remdesivir for moderate infections was favoured in only 0.001% of simulations.

**Figure 2:**
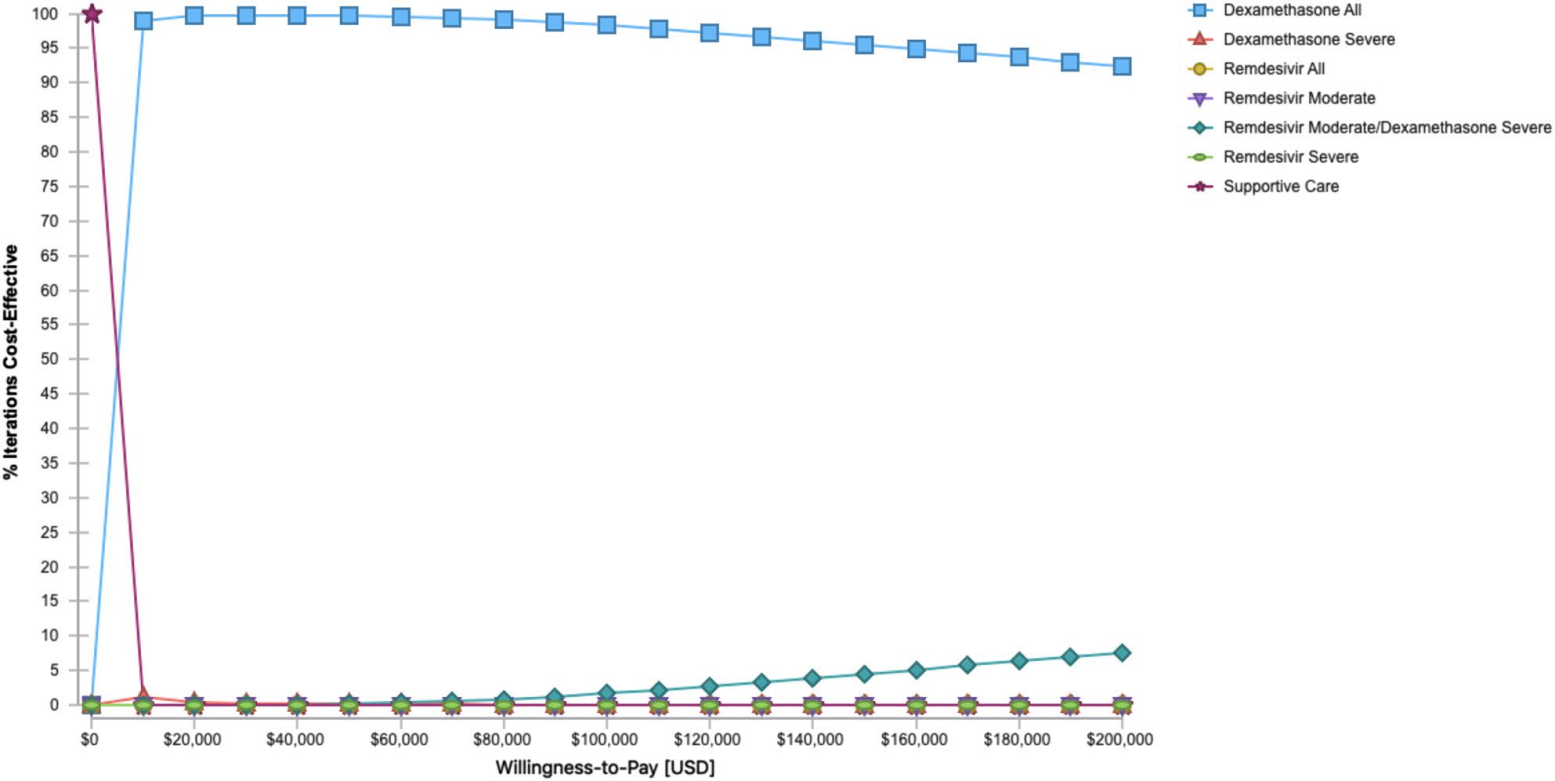
Cost effectiveness acceptability curve with probabilistic sensitivity analysis. Analysis of likelihood of a strategy being preferred based on the willingness to pay threshold in 2020 US dollars. Dexamethasone for severe infections, remdesivir for moderate infection and remdesivir for all overlap at bottom.

The cost effectiveness acceptability curve (Fig. 2) shows that as the willingness to pay threshold increases remdesivir for moderate and dexamethasone for severe infection strategy became more favoured although significantly less than that of dexamethasone for all patients. Conversely, with willingness to pay thresholds lower than the typical US standard of $100,000/QALY, the use of dexamethasone for only severe COVID-19 infections would be favoured with a willingness to pay threshold between $250-$1250/QALY while supportive care would be favoured with a willingness to pay threshold of less than $250/QALY.

## Discussion

In our base-case analysis, we found that using dexamethasone for all patients the most cost-effective strategy to treat moderate-severe COVID-19 infections with a cost of $980.84/QALY per person per year as compared to supportive treatment. All strategies using remdesivir were less effective and more costly than other strategies in the base-case. Probabilistic sensitivity analysis showed that dexamethasone for all patients remained the preferred choice when willingness to pay thresholds are over $1250 USD/QALY.

Given the significant morbidity and mortality of moderate-severe COVID-19 infection, vaccine distribution is critical. In the interim, any treatment that may improve outcomes is valuable but must be balanced with treatment affordability and health care sustainability given the opportunity cost associated with use of these drugs. This model compares the first two agents with randomized controlled trial data showing potential mortality benefit in moderate-severe COVID-19 infections, with remdesivir showing a statistical trend to survival in the preliminary report with a larger effect in patients with moderate infections and dexamethasone demonstrating a statistically significant benefit in both moderate and severe infections.

The model design uses a fixed cost for admission with the DRG code and does not account for potential shorter stays in hospital and does not include physician fees. Given our use of the payer’s perspective, the cost of the hospital stay will be the same rate based on the DRG code regardless of the length of stay. DRG based systems are designed to provide a package price for a bundle of care based on acuity and increase efficiency of care^19^. These are used in much of Europe^20^ and a number of Asian Pacific countries^19^. Impacts on the length of stay would affect physician fees which are approximately $100/day for the ward and $230/day for the ICU^21^ which are all less expensive than one dose of remdesivir. Given data suggesting remdesivir might shorten^5^ or lengthen^22^ hospitalization, the impact on length of stay requires further study. Although this model is most directly appliable to DRG using countries, DRG rates reflect the average cost for diagnoses in the group^20^ and so are a reasonable estimate of cost; as such, the model outputs can reasonably be extrapolated to other countries that are based on a fee-for-service model.

In our base case scenario, we assumed that moderate COVID-19 infections would be admitted to the ward and severe infections admitted to the ICU. This practice is not consistent throughout the United States; some centers will admit patients on high flow oxygen to ICU which would subsequently increase costs. In our analysis of this scenario where patients with both moderate and severe intensity COVID-19 were admitted to ICU, we found that the league table and ICER values between strategies remain unchanged, but all strategies would cost an additional $22,134.

The economic and health impact of COVID-19 has been substantial globally^23^; treatments that can reduce its burden are eagerly sought. Use of either dexamethasone or remdesivir for COVID-19 needs to be considered in the context of the local burden of COVID-19 disease as well as healthcare budgets and priorities. Decisions of treatment and resource utilization need to be made rationally and with consideration of the values and priorities of the population. Although our representative analysis utilized a United States perspective, we feel that these results can be extrapolated to other jurisdictions world-wide, as the major cost for each strategy was hospitalization; with lower costs of hospitalization, the incremental cost-effectiveness ratios between choices will remain similar given all strategies would have the same reduction in total cost as demonstrated in a scenario analysis. The preferred strategy for each country is driven by opportunity costs and each jurisdiction’s priorities, budgets and willingness to pay.

Thresholds for cost-effectiveness worldwide vary; in the United States, the threshold is typically $100,000^16^ while the threshold is generally considered to be around $50,000 CDN ($36,784 USD)^24^ in Canada and between 20,000 and 30,000 pounds ($25,245-$37,868 USD)^25^ in the United Kingdom. For lower income countries, the cost-effectiveness threshold is markedly lower ranging from $3-$8982 (2020 US dollars)^26^. In our analyses, we show that for countries of low to middle income, with willingness to pay thresholds over $1250, dexamethasone for all patients would likely be the most favoured strategy based on current data.

An important factor to be considered in addition to cost-effectiveness is the impact of a treatment strategy on the budget of a jurisdiction. Cost-effective treatment in most cases increases expenditures; treatments that are more effective and cost saving are extremely uncommon. Even if treatments are cost-effective and relatively inexpensive, such as dexamethasone for patients with moderate-severe COVID-19, high burden of disease may have an extremely significant impact on the budget. As such, for very low-income countries, the price of dexamethasone may be unaffordable and so supportive care would be the preferred strategy.

COVID-19 has had disproportionate impacts on patients in the United States based on ethnic background, location and socioeconomic status. Patients of colour including African Americans, Latinx and Native Americans, immigrants, patients in rural settings and patients with lower socioeconomic status in the United States have had increased morbidity and mortality due to COVID-19^27–29^. Similar findings have been reported worldwide, especially in countries of lower socioeconomic status^30^. Although dexamethasone is relatively inexpensive, newer treatments for COVID-19 that may be more effective will likely be more costly. Ensuring access to effective and affordable treatment for COVID-19 is important as lack of access will likely further negatively impact people of colour, rural patients or patients with lower socioeconomic status.

There has been one published cost-effectiveness analysis of remdesivir looking at cost-effective threshold prices finding that a treatment course of remdesivir should be approximately $19,000 to be cost effective based on a willingness to pay of $100,000 and about $4700 based on a willingness to pay of $50,000, suggesting that a cost recovery price should be between $1000–1600^31^. This model takes a lifetime perspective and uses remdesivir for all patients. Our model has a few key differences. We have taken a 1-year time-horizon, used severity-stratified drug efficacy based on current clinical trials data to better reflect the differences between moderate and severe infections, and utilized Medicare rates which may underestimate costs in the United States. In our analysis, we directly compare remdesivir versus dexamethasone for all hospitalized patients, as well as remdesivir for moderate and dexamethasone for severe infections to reflect current pragmatic treatment approaches based on the two available trials; we do not combine dexamethasone and remdesivir as treatment as there is no data for this.

There are several limitations to our model. First, our model and base case is based on the available literature with relatively limited treatment randomized controlled trial outcome data available; this data has been extrapolated to make treatment recommendations for the general population^32^. Given that COVID-19 is an emerging disease with rapidly evolving literature, the assumptions in the model are subject to change. Data regarding utility in COVID-19 does not yet exist and was extrapolated from similar experience with H1N1 and severe influenza. Our hospital costs were based on the Medicare price; in other centers, rates may be higher with private insurance. We did not account for differences for length of stay with treatment or physician fees which would impact the model; given that remdesvir’s impact of length of stay is unclear^22^ and the largest component of cost would be hospitalization proper, we feel the impact would not impact the overall conclusion. Last, we assumed that beyond the initial 28 days, there would be no further impact to health utility and mortality. Given COVID-19 was only first described in December 2019, 1-year data is only becoming available about outcomes including the uncertain impact of ‘long COVID’^33^ on health utility. We do not account for potential adverse impacts of medication including remdesivir and dexamethasone; adverse effects would reduce the utility scores of the strategy and given it is unlikely that the adverse effects of dexamethasone are markedly worse than remdesivir, we feel that the overall ranking of strategies would not be significantly different, but the cost-effectiveness ratios may slightly vary. Further, although there are some data regarding effects post infection^34^, the impact of COVID-19 after the initial infection is still to be determined. To mitigate the uncertainty, we performed a probabilistic sensitivity analysis where the estimates of costs, utilities and probabilities were varied simultaneously over their distributions and found that dexamethasone for all patients remained the preferred strategy. This model does not combine use of dexamethasone and remdesivir in individual patients as there is no published data for this strategy.

In summary, use of dexamethasone for all patients with moderate-severe COVID-19 emerged as the most cost-effective management, and dexamethasone for severe infections was favoured with lower willingness to pay thresholds. Additional information about the effect of remdesivir is required to better assess the cost-effectiveness of its use although at this time, the economic argument for its use is difficult to make.

## Data Availability

Data inputs used for the model are available in Table 1.
